# Urochordate Histoincompatible Interactions Activate Vertebrate-Like Coagulation System Components

**DOI:** 10.1371/journal.pone.0003123

**Published:** 2008-09-01

**Authors:** Matan Oren, Marie-line Escande, Guy Paz, Zvi Fishelson, Baruch Rinkevich

**Affiliations:** 1 Israel Oceanographic and Limnological Research, National Institute of Oceanography, Haifa, Israel; 2 Université Pierre et Marie Curie-Paris 6, Laboratoire Arago, Avenue Fontolé, BP44, Banyuls sur Mer, France; 3 Department of Cell and Developmental Biology, Sackler School of Medicine, Tel Aviv University, Tel Aviv, Israel; Centre de Recherche Public-Santé, Luxembourg

## Abstract

The colonial ascidian *Botryllus schlosseri* expresses a unique allorecognition system. When two histoincompatible *Botryllus* colonies come into direct contact, they develop an inflammatory-like rejection response. A surprising high number of vertebrates' coagulation genes and coagulation-related domains were disclosed in a cDNA library of differentially expressed sequence tags (ESTs), prepared for this allorejection process. Serine proteases, especially from the trypsin family, were highly represented among *Botryllus* library ortholgues and its “molecular function” gene ontology analysis. These, together with the built-up clot-like lesions in the interaction area, led us to further test whether a vertebrate-like clotting system participates in *Botryllus* innate immunity. Three morphologically distinct clot types (points of rejection; POR) were followed. We demonstrated the specific expression of nine coagulation orthologue transcripts in *Botryllus* rejection processes and effects of the anti-coagulant heparin on POR formation and heartbeats. *In situ* hybridization of fibrinogen and von Willebrand factor orthologues elucidated enhanced expression patterns specific to histoincompatible reactions as well as common expressions not augmented by innate immunity. Immunohistochemistry for fibrinogen revealed, in naïve and immune challenged colonies alike, specific antibody binding to a small population of *Botryllus* compartment cells. Altogether, molecular, physiological and morphological outcomes suggest the involvement of vertebrates-like coagulation elements in urochordate immunity, not assigned with vasculature injury.

## Introduction

Blood coagulation, a vital body defense mechanism [1], is a shared vertebrata mechanism that allows an improved management of blood fluidity following vascular injury. This ancient complex process [2], by which cells and blood-borne molecules develop solid clots, controls blood loss from damaged vessels, as part of hemostasis.

The hemostatic feature of coagulation is activated immediately upon injury, as damage to blood vessel walls exposes collagen (normally present under the endothelium), to which circulating platelets bind through specific surface receptors. This adhesion is further strengthened by the von Willebrand factor (vWF) circulating protein, which forms links between platelet glycoproteins and collagen fibrils, ensuring the formation of primary hemostatic plug. Then, to assure the stability of the primary hemostatic plug, the platelets stimulate local activation of plasma coagulation factors, leading to generation of a fibrin clot that traps and reinforces the hemostatic plug [3]. A characteristic of vertebrate coagulation and hemostasis is the thrombin-generated fibrin clot. Thrombin, the key proteolytic enzyme, presides over the conversion of the soluble plasma glycoprotein fibrinogen into fibrin monomers. These monomers spontaneously aggregate, forming, in conjunction with the platelets, the mesh of the hemostatic fibrin clot. In search for the evolution of vertebrate blood coagulation Davidson et al. [2] draws a 450 million year old hemostasis system that had evolved during the 50–100 million year period separating the appearance of urochordates and vertebrates from their common ancestor [4]. Whereas the chordate *Amphioxus* has a thrombin-like enzyme but no true fibrinogen [5], the solitary urochordate *Ciona intestinalis* genome did not display any genuine orthologues for the coagulation genes, although paralogues and constituent domains had been recorded for virtually all coagulation factors [4]. This analysis confirmed earlier studies on the solitary ascidian *Halocynthia roretzi*, suggesting the absence of the vertebrates' coagulation system in its hemolymph [6], while featuring a thrombin-like serine protease that can convert mammalian fibrinogen to fibrin, producing a clot [7].

The above notion is further strengthened by studies on other invertebrates. One most relevant example [8] revealed that the well-characterized clotting cascade of the horseshoe crab *Tachypleus tridentatus* is linked to the activation of the nonself-recognizing cascade, prophenoloxidase system, with the oxygen carrier hemocyanin functioning as a substitute for prophenol oxidase. This hemolymph coagulation system is strongly activated by bacterial elicitors, in contrast to vertebrate coagulation where induction relies primarily on signals from damaged endothelial tissue. It is also evident that invertebrates like arthropods use different enzymes to crosslink the clot compared to vertebrates, and that arthropod hemolymph clotting factors are not orthologues of vertebrate blood clotting factors [9].

Innate immunity in *B. schlosseri*, as in other botryllid ascidians, expresses a unique allorecognition system, in which genotypes that do not share any allele on the highly polymorphic histocompatibility haplotype, the Fu/HC locus [10] reject each other. Allogeneic rejections in *B. schlosseri* start with the accumulation of granular cells, termed “morula cells”, in the interacting vasculature tips (ampullae) and followed by partial fusion of the cuticle between interacting colonies [11]. Morula cells contain inactive prophenoloxidase inside intracellular vacuoles that becomes activated during the process [12], producing melanin, which accumulates as brownish color dots in confined areas of the tunic between interacting ampullae. These areas, termed “points of rejection” (PORs) consist of clumps of dead blood cells, mainly of the morula type.

To date, no vertebrate-like coagulation system has been reported in the hemolymph of non-vertebrate chordates or other invertebrate animals [4], [13]. It was therefore surprising to find a prominent group of coagulation genes in the cDNA library of differentially expressed sequence tags (ESTs), developed for allorejection processes in the colonial urochordate *Botryllus schlosseri* [14]. A detailed analysis of the library ESTs disclosed a comprehensive list of coagulation candidates. The list includes orthologues of thrombin, thrombin inhibitors, von Willebrand factor, coagulation like serine proteases and ESTs resembling or containing motifs of fibrinogen and coagulation factors V and VIII. The discovery of coagulation-related sequences in the allo-rejection EST library of a colonial tunicate, together with POR formation that morphologically resembles a vertebrate clot, led us to test whether a vertebrate-like clotting system is being activated during *Botryllus* allorejection as an integral part of its innate immune system.

## Methods

### Allorecognition assay

We used laboratory-bred *B. schlosseri* colonies, offspring of founders collected at Monterey, Half Moon Bay and Moss Landing, CA, USA, and Banyuls, France marinas. The animals were maintained on glass slides at 20°C, in a flow-through facility at the National Institute of Oceanography, Haifa as described [15]. Large colonies were routinely subcloned into ramets of different sizes that were then transferred onto other glass slides, for further growth. Hence, the same genotypic constituent could be simultaneously used for both control and repeated assays. Allorecognition assays [16] were performed on paired histoincompatible colonies juxtaposed on a glass-slide at a distance of less than 1 mm in order to allow fast contact between extending peripheral ampullae.

### Heparin administration assays

Two assays were performed with heparin sodium (Pharmacare Ltd., South Africa) in order to check its impact on histoincompatible reactions. Assay #1 analyzed low doses (100 units/L) impacts. Control and heparin treatments were carried out in 1.5 L plastic containers of seawater at 20°C. We examined 12 different histoincompatible pairs in six separate experiments. Identical genetic pairs were used as controls in seawater containers without heparin. Heparin was administrated into the water and was replaced every 48 h. Observations were done every 24 h, for 5 days, under a stereomicroscope (×100 power).

In assay #2, we used high doses of heparin sodium (up to 40,000 units/L) in order to check impacts on *Botryllus* heart beat rates. For this experiment, we chose actively filtering colonies. Heparin treatments were carried out in Petri dishes containing 50 ml of seawater at 20°C. Ten minutes after heparin administration, the medium was replaced with fresh seawater. Heartbeats per 10 seconds were counted every five minutes for the same heart.

### Light and electron transmission microscopy

Histological preparations were made from tissue samples of interacting and naïve ampullae that were fixed in 4% formaldehyde for 2 h, embedded in paraffin, serially cross-sectioned (5 µm) and stained by hematoxylin and eosin. Observations were performed under Olympus BX50 Upright microscope, equipped with Color View camera (Soft Imaging System, Munster, Germany).

For EM analyses, naïve and allogeneic interacting subclones were fixed in 2.5% glutaraldehyde in seawater and stored at 4°C for 10 days. Next, they were washed thrice in 0.2M cacodylate buffer (pH 7.2–7.4) and postfixed by 0.1% OsO_4_ in the same buffer for 1 h, at room temperature. After three buffer washes, samples were dehydrated by ethanol series and embedded in Epon 812. Zones of interest were selected and positioned properly in flat embedding molds. Ultrathin sections of 80 nm were cut with Leica Ultracut R ultramicrotome. Finally, the sections were stained with uranyl acetate and lead citrate. Grids were observed under a Hitachi 7500 electron microscope. Alternatively, thin sections of 1 µm were stained by a mixture of toluidine blue and azur II and observed under a Leica DME photonic microscope.

### RNA extraction and RT-PCR

Total RNA was extracted, separately, from interacting pairs and from corresponding naïve ramets (n = 4) by EPICENTER MasterPure™ RNA Purification kit. The integrity of the total RNA was verified by agarose gel electrophoresis. To verify differential expression of coagulation related ESTs we chose nine EST transcripts from the *B. schlosseri* rejection EST library for RT-PCR reactions. First strand cDNA was synthesized by DNA synthesis kit (Fermentas, MD, USA). The PCR amplification was performed using designed sets of primers (Metabion, Martinsried, Germany) as depicted in Table 1. The PCR reaction was carried out for 24 cycles (94°C, 45 sec; 55–61°C, 45 sec; 72°C, 1 min) followed by additional 5 min at 72°C. We used *Botryllus* actin (Accession No. AY159281) as reference gene with the same parameters as noted above. Four microliters of each PCR product was analyzed in 1.2% agarose/EtBr gel alongside DNA marker.

**Table 1:** Primers used for RT PCR.

EST id	5′ primer			3′ primer			Annealing Tmp.
Contig182	5′	TACGCTCCAGAACGACATCATG	3′	5′	AACATCGGCGTAAACACCTGG	3′	61°C
Contig179	5′	CCTTTCCGTTGACGCTGATAG	3′	5′	CATTACCACGTGAACGACAAC	3′	59°C
M21_H4	5′	CAAAGAGTTAGGAGTGTCGCTG	3′	5′	GCGTATGCAATGAATGTGAAG	3′	57°C
M20_E3	5′	GACCAGATGTCGCCAGGACTTG	3′	5′	TGACGAGTTGTTCTCCCTCCTC	3′	55°C
Contig68	5′	AATATCATCCATGTCAAGCTGC	3′	5′	TCTTCCGGGCTACAGTCATATC	3′	58°C
M19_B3	5′	CGTCTACCGCCGTCTTTAG	3′	5′	AACACCATCGTCGAGAACTTC	3′	59°C
M2_E7	5′	CAATTTCGGCTCCAAGAGTCG	3′	5′	CGAAGCTGTGCAATGCAACG	3′	60°C
contig144	5′	CAGCGAAGAATGAATGGAGAC	3′	5′	AGTTTCCGTTCAGGTTGCTG	3′	58°C
M21_G10	5′	ACGTAAGTTTGAATGCCAGGTC	3′	5′	GGCACGATCACCTACACCAG	3′	60°C
BS cytoplasmic actin (Ref gene)	5′	GTAGGTAGTCTCGTGAATTC	3′	5′	CACGCCATCTTGCGTCTGGA	3′	57.2°C

### TUNEL assay

Apoptotic nuclei were stained using a Klenow fragEL DNA fragmentation detection kit (TUNEL) (QIA21, Calbiochem, Germany) on paraffin-embedded tissue sections, according to the manufacturer's protocol. Negative controls were generated by substituting the Klenow enzyme in the labeling reaction mix with dH_2_O.

### *In-situ* hybridization

Interacting histoincompatible colonies and their naïve counterpart clones were fixed overnight in 4% paraformaldehyde, dehydrated in 70% methanol, embedded in paraffin and cut into 5 µm sections. EST clones were used to obtain sense and antisense DIG-labeled RNA probes that were synthesized with the DIG RNA Labeling Kit (SP6/T7; Roche Molecular Biochemicals, Mannheim, Germany). Hybridization of probes to tissue sections was performed according to Breitschopf et al. [17] for paraffin-embedded tissues. DIG-labeled RNAs on samples were observed using anti-DIG antibody (Roche).

### Immunohistochemistry

Immunohistochemistry was performed on histological sections as described by Lapidot et al. [18] using alloreactive naïve and interacting colonies. We used goat anti-human fibrinogen antibody (F-2506, Sigma Aldrich, Israel) as the first antibody at a dilution of 1∶2500 and Cy-2-labeled rabbit anti-goat antibody (Jackson ImmunoResearch labaratories inc., PA, USA) as secondary antibody at a dilution of 1∶250. The slides were mounted with Fluoromount-G glue (Southern Biotech., AL, USA) and kept at 4°C until observation.

## Results

### Clot formation during allo-rejection responses

**Figure 1:**
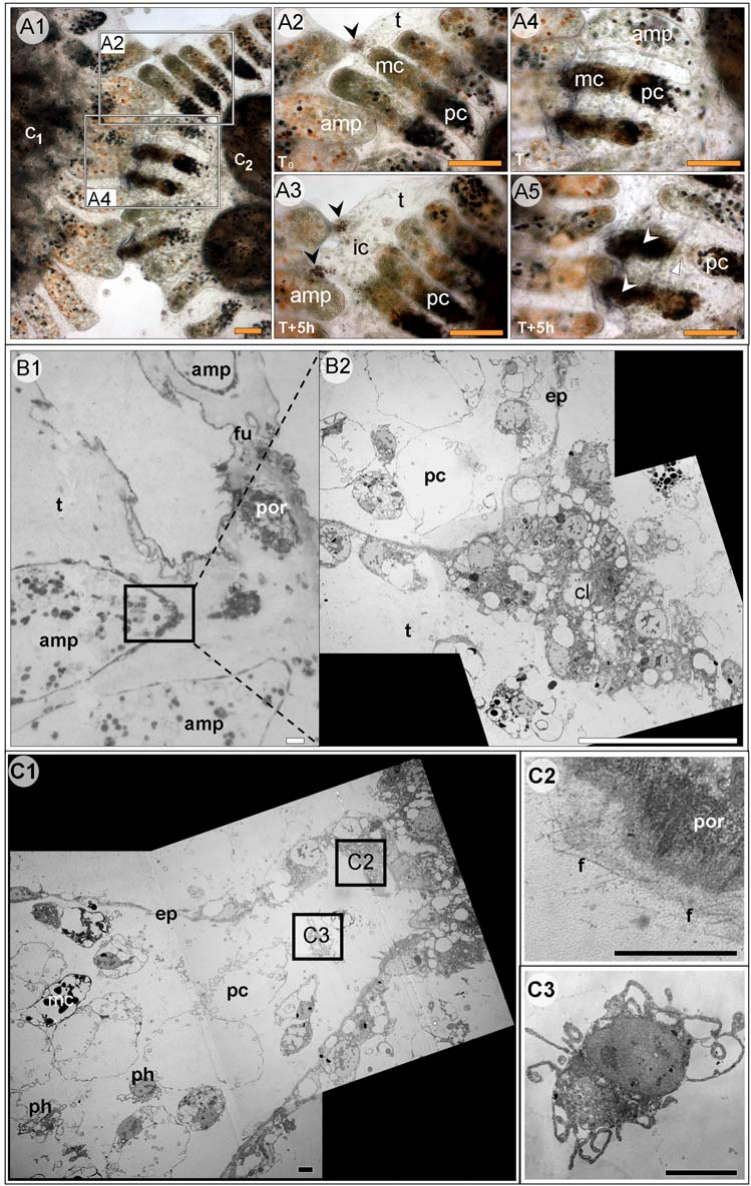
Botryllus schlosseri types I, II and III rejections. (A1) Allogeneic incompatible pair of colonies featuring both I and, II rejection types (A2, A3 and A4, A5 respectively). (A2) Type I rejection at onset, with morula cells concentrating at the ampullae tips and initial infiltrations toward the newly formed POR (black arrowhead). (A3) After 5 h, most of the morula cells are already outside of the ampullae. Ampullae shrink and retreat, leaving behind two PORs. (A4) Type II rejection at onset, morula cells are concentrating at the ampullae tips. (A5) After 5 h, one ampulla is amputated (white triangular). Clumps of activated dark morula cells are formed within ampullae lumens (white arrowheads). (B, C) EM of type III rejection (B1) A general overview of type III rejection (B2) Enlargement of a ampulla tip area; Blood cells clot seals the epithelium gap, created during type III rejection (C1) a ruptured ampulla with enlarged areas revealing: (C2) Ultramicroscopic fibers scattered along the inner margins of the clot (C3) A phagocyte in type III interacting ampulla. amp- ampulla, c_1_- colony 1, c_2_ - colony 2, cl- cellular clot, ep- epithelium, f-fibers, fu- fusion area, ic- infiltrated cells, mc- morula cell, pc- pigment cell, ph- phagocyte, por- point of rejection, t- tunic. Orange scale bar: 100 µm (A1–A5), white scale bar: 20 µm (B1–B2), black scale bar: 2 µm (C1–C3).

**Figure 2:**
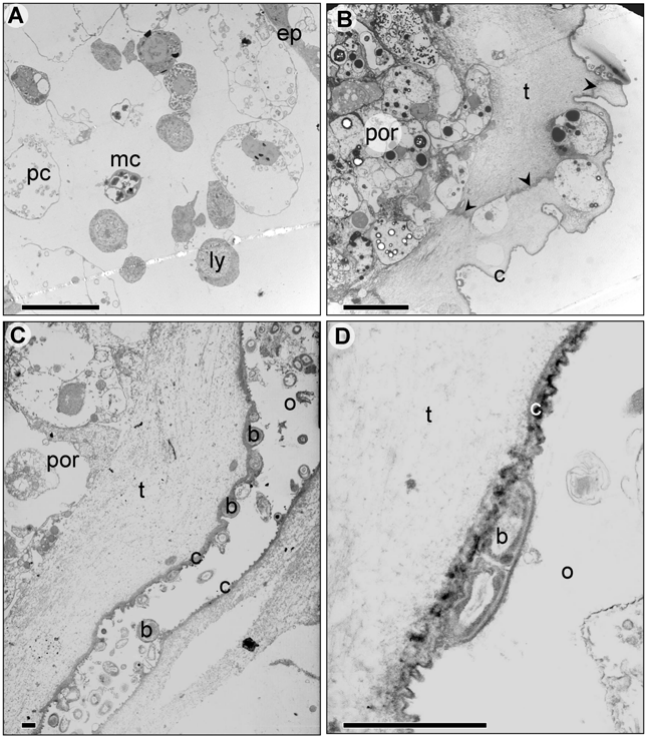
General characteristics of *Botryllus* allo-rejection areas, as revealed by EM. (A) Part of a POR surrounded by tunic in the area between interacting colonies. Black arrowheads indicate fibrous ‘threads’ inside the tunic along which the colonies will be separated. (B) Cells inside lumen of an interacting ampulla. (C, D) Bacteria attached to the outer cuticle surrounding the POR, some at cell division (D). b- bacteria o- outer surrounding c- cuticle, ep- epithelium, ly- lymphocyte-like cell, mc- morula cell, pc- pigment cell, por- point of rejection, t- tunic. Scale bars: 10 µm (A, B), 1 µm (C, D).

**Figure 3:**
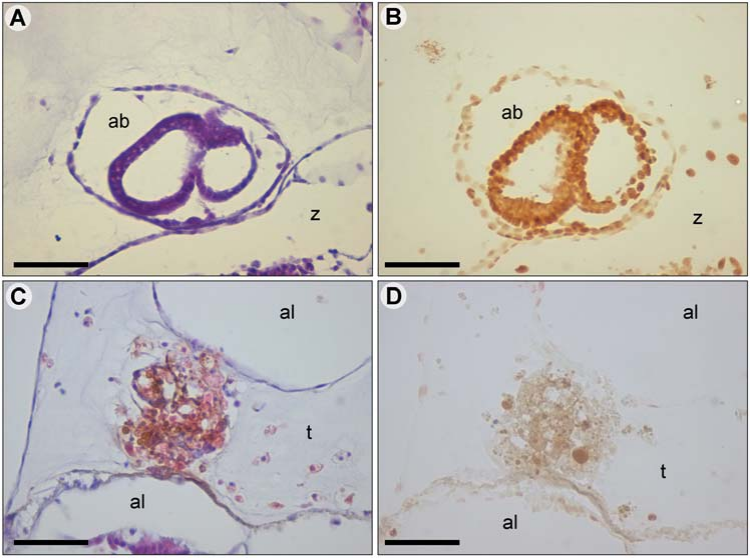
TUNEL staining of *Botryllus* during allorecognition response and blastogenesis. (A) An H&E stained apoptotic secondary bud at early developmental stage. Usually buds are maturated to be fully functional zooids during *Botryllus* weekly blastogenic cycle. In few cases, as in this case, buds do not achieve maturation and are going through apoptosis. (B) A subsequent section to (A): HRP stained fragmented DNA in the majority of bud cells using Tunel. (C) H&E stained point of rejection (POR). (D) A subsequent section to (C). HRP stained POR using TUNEL. Only few cells are stained. ab- apoptotic bud, al- ampulla lumen, por- point of rejection, t– tunic, z- zooid. Scale bar: 50 µm.

*B. schlosseri* histoincompatible reactions (rejections) were classified into three major types (I–III), according to their distinct histological morphologies, akin to a previous suggestion [11], [19]. To avoid variations in the results caused by different onsets of rejection patterns and genotypic specific reactions, rejection classifications were established by analyzing histological sections done on the climax status of the developed POR. Type-I rejection exhibited the mildest rejection presented by the allogeneic challenged ampullae, with morphologically intact, undamaged epithelium. In this rejection type, blood cells (mostly morula cells) infiltrate through the ampulla epithelium tip toward the colonies' encountered borderlines, where several small PORs are formed (Fig. 1, A1–A3). Type-II rejection is characterized by amputation of ampullae from vasculature at ampulla-blood vessel link points. This amputation is not associated with excessive bleeding, or hemorrhage formation. The amputated ampullae were loaded with activated morula cells that were clumped together to form PORs in the size of the ampullae tips (Fig. 1, A4, A5) and resulted in larger POR compared to Type-I rejection. Type-III rejection started with ampulla tips' rupture and blood cells leakage through the shattered epithelium. Then, blood cells at the tip of the ruptured ampullae adhered together thus sealing the newly formed wounds in the epithelium (Fig. 1, B1, B2). This cell aggregates eventually became a part of the POR. TEM observations disclosed ultramicroscopic fibers associated with this newly formed clot, similar to observations made by Scofield and Nagashima (11; Fig. 1, C1, C2). In type-III rejections, the ruptured ampullae were populated by phagocytes (Fig. 1, C3), in addition to other circulating blood cells, mostly morula cells, pigment cells, and lymphocyte-like cells (Fig 2, A). It is not uncommon to find more than one type of rejection simultaneously developing side by side in a specific allogeneic pair. TEM sections performed on rejecting *Botryllus* ampullae indicated that POR clots are primarily made of necrotic morula cells in an advanced state of disintegration containing electron dense vacuoles in various sizes. The cells in a POR clot are tightly adhered to each other with almost no space between them, whereas the surrounding transparent tunic milieu turns into a fibrous matrix that develops several extensively deposited fibrous ‘threads’ that cross the tunic between the PORs edges and the tunic cuticle (Fig. 2, B). These fibrous threads were found to be associated, in other botryllid ascidians, with the dissociating borders between interacting allogeneic partners, physically separating the interacting colonies [20], [21]. After separation, the clot area in the colonial periphery detach from the rest of the colony's body and degenerate as the tunic cuticle, which becomes heavily populated by bacteria (Fig. 2, C, D). TUNEL assays performed on rejecting areas resulted in almost no positively stained cells at the POR, compared to massive apoptosis in *Botryllus* colony buds (Fig. 3, A–D), thus negating the possible involvement of apoptosis during the rejection process and clot formation.

### Heparin affects POR formation and heartbeats

**Figure 4:**
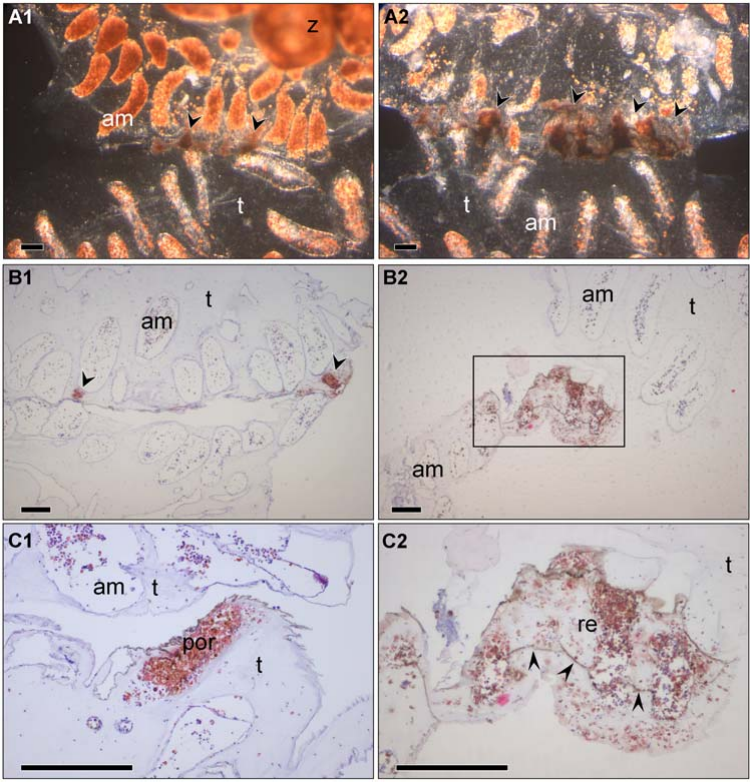
Heparin impacts on allogeneic interacting *Botryllus* colonies. (A_1_–C_1_) Control non-treated rejecting pairs. (A_1_) Ctrl rejection *in vivo*. Arrowheads indicate the sites of developed PORs along the border between the interacting colonies. (B_1_) H&E stained histologoical section of a non-treated rejecting pair. PORs are condensed in limited areas (arrowheads) of the tunic. (C_1_) H&E stained section; Enlargement of a typical POR, formed during non-treated rejecting reaction. (A_2_–C_2_) Identical genetic rejecting pairs treated with 100 units/L heparin sodium. (A_2_) Heparin treated rejection *in vivo*. Arrowheads indicate the sites and sizes of the rejection areas (B_2_) H&E stained histologoical section of heparin effected rejecting pair. No obvious POR, but a larger rejection area (inside the marked rectangle) instead. (C_2_) H&E stained section; Enlargement of heparin effected rejection area. Blood cells are sporadically spread over a wide area inside the tunic between the interacting colonies. am-ampulla, por- point of rejection, re- rejection area, t- tunic, arrow heads indicate dissociation lines. scale bar: 100 µm

**Figure 5:**
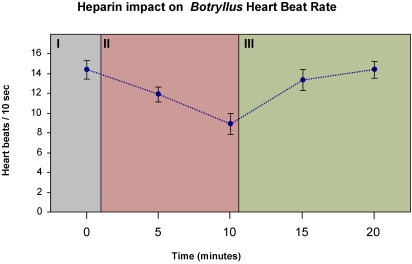
Heparin affects *Botryllus* heartbeat rates (n = 10). Phase I. Normal heartbeats, averaging 14.4 beats per 10 seconds. Phase II. Ten minutes following administration of 40,000 units/L heparin sodium. Heartbeats decreased to an average 8.9 beats per 10 seconds. Phase III. Heartbeats full recovery within 10 minutes from changing the medium.

Administration of 100 units/L heparin sodium in 1.5 L tanks holding *Botryllus* rejection pairs (N = 12) led, in 25% of the cases, to different morphological features than seen in types I–III rejections developed in control ramets. Very intense and large debris with indistinguishable borders and highly distinctive conglomerations of melanized degraded cells characterized areas of rejection in these interactions (Fig. 4, A_2_). In histological sections of non treated rejecting colonies, eosin stained cells were typically found to be concentrated inside the PORs (Fig. 4, B_1_, C_1_), whereas in 25% of the histological sections after heparin treatment, eosin stained cells were sporadically spread over a large area and across the tunic separating between the interacting colonies (Fig. 4, B_2_, C_2_). In contrast to regular rejections, heparin effected rejections exhibited no cell clots along the fused tunic borderlines although cells underwent melanization (Fig. 4, A_2_). For physiological impacts of heparin, we administrated increasing doses of heparin to the water. At concentrations of 40,000 units/L we noticed a 38% decrease (N = 10; Before: 14.4±0.97; After: 8.9±1.1) in the average heartbeat rates within 10 minutes after heparin administration (Fig 5; Video S1, S2). At times, the heartbeat rates became irregular. A complete recovery of the heartbeats was observed within less than 10 minutes after removal of the heparin (Fig. 5). We further monitored the treated colonies for several weeks after recovery and observed no permanent damage or physiological changes.

### Differential expression of coagulation-related genes during *Botryllus* allo-rejection

A blast analysis for *Botryllus* rejection subtraction library [14] identified several candidate coagulation proteins and coagulation-related protein families. In total, 34 protein matches were categorized as cell adhesion and coagulation orthologues. This category included five entries of the key vertebrate coagulation gene von Willebrand Factor (vWF) and several members of the selectins (P and E), mucins and annexins protein families. Two other prominent groups expressed in the library were the serine proteases group, including genes of the trypsin family, and the serine protease inhibitors (serpins) group. While re-screening the library in search for coagulation orthologues, we identified the following new blast matches: thrombin (acc. number: FF339595), milk fat globule-EGF factor 8 with high similarity to coagulation factor V (acc. number: FF339598), ovochymase 2 with high similarity to coagulation factor IX (acc. number: FF339596), connective tissue growth factor (acc. number: FF339597) and tissue factor pathway inhibitor 2 (acc. number: FF339642; Table 2). Additionally, four ficolin orthologues were identified (with lower similarity) as fibrinogen/fibronectin orthologues; AsMASPb orthologue was also identified as plasminogen orthologue; *Botryllus* serine protease orthologues were also identified as chymotrypsinogen orthologues and the serine protease inhibitor orthologues were also identified as thrombin inhibitor orthologues (Table 2).

**Table 2:** Major candidate coagulation genes found in *Botryllus* rejection EST library.

EST ID	Protein match name	organism	Accession No.	E-value	Other relevant matches	E-value
M1_A7	Thrombin	*G. gallus*	FF339595	3E-04	—	—
M21_H4	von Willebrand Factor like 1	*C. intestinalis*	EE743653	6E-40	—	—
M20_E3	*	*	EE743654	1E-39	—	—
M23_D7	Vwa1 protein	*B. villosa*	EE743538	3E-06	—	—
M7_G8	*	*	EE743539	1E-10	—	—
Contig182	Trypsin	*P. misakiensis*	EE743601	2E-73	—	—
Contig179	Trypsinogen	*B. schlosseri*	EE743603	3E-43	—	—
M3_B6	*	*	EE743604	9E-19	—	—
M18_B11	*	*	EE743605	1E-33	—	—
Contig177	Trypsinogen	*B. schlosseri*	EE743606	8E-37	—	—
Contig22	Chymotrypsinogen	*B. villosa*	EE743607	2E-16	—	—
M2_C9	*	*B. villosa*	EE743608	2E-20	—	—
M11_F4	Alpha-1-antitrypsin-like protein	*T. sibiricus*	EE743621	2E-05	Antithrombin III [*M. domestica*]	3E-04
M7_G3	Serpin 1 precursor	*B. lanceolatum*	EE743622	6E-20	Thrombin inhibitor [*B. taurus*]	5E-16
M7_A4	Serpin B1	*E. caballus*	EE743625	1E-29	Thrombin inhibitor [*B. taurus*]	4E-23
M5_A7	Tissue factor pathway inhibitor 2	*D. rerio*	FF339642	9E-18	—	—
M17_D9	Connective tissue growth factor	*M. musculus*	FF339597	2E-05	—	—
M12_D5	AsMASPb	*H. roretzi*	EE743594	3E-33	Plasminogen [*B. taurus*]	2E-08
M22_F8	coagulation serine protease	*C. intestinalis*	EE743599	1E-10	—	—
M20_F12	Serine protease	*B. schlosseri*	EE743595	1E-18	Chymotrypsinogen B1 [*X. tropicalis*]	9E-15
Contig121	*	*	EE743596	2E-21	Chymotrypsinogen 2 [*X. tropicalis*]	8E-11
M23_H5	*	*	EE743597	5E-09	Chymotrypsinogen B1 [*B. Taurus*]	2E-06
Contig85	*	*	EE743598	9E-23	Chymotrypsinogen A [*G. gallus*]	4E-11
M2_E7	Ficolin 2 precursor	*H. roretzi*	EE743582	4E-45	Fibrinogen and fibronectin [*C. pipiens*]	5E-35
M6_H11	*	*	EE743583	4E-24	Fibrinogen and fibronectin [*A. aegypti*]	2E-19
contig144	Ficolin 4	*H. roretzi*	EE743580	2E-42	Fibrinogen and fibronectin [*A. aegypti*]	1E-34
M4_G8	Ficolin 3	*H. roretzi*	EE743581	2E-36	Fibrinogen [H. sapiens]	1E-29
M11_F3	Ficolin	*S. scrofa*	EE743584	3E-11	Fibrinogen and fibronectin [*C. pipiens*]	3E-04
M21_G10	Thrombospondin 2	*H. sapiens*	EE743592	9E-07	—	—
M22_G7	Ovochymase 2	*M. musculus*	FF339596	5E-08	Coagulation factor IX [*D. rerio*]	2E-06
Contig68	Milk fat globule-EGF factor 8	*X. tropicalis*	FF339598	7E-10	Coagulation factor V [*P. textilis*]	2E-07

The ESTs from *Botryllus* rejection library were subjected to protein motif analysis against InterPro database, using InterProScan software (local installation). Based on InterPro parceling, 47 different coagulation-related domains were identified (E-Value≤0.001). This includes five coagulation factor 5/8 domains, seven fibrinogen domains, four von Willebrand factor (type A and D) domains, three thrombospondin domains, four protease inhibitors I4 and I8 (serpin) domains, 15 peptidase S1 and S6 chymotrypsin/Hap and trypsin-like serine and cystein domains, five EGF-like and EGF calcium-binding domains, aspartic acid and asparagine hydroxylation site, transglutaminase and MAM domains (Table 3).

**Table 3:** Library coagulation domains.

Motif name	InterPro ID	Rep. no.	Seq Id.	E-Value	GeneBank Acc. No.
Coagulation factor 5/8 type, C- Terminal	IPR000421	1	M19_B3	3E-12	FF339599
		2	M3_B4	2E-08	FF339600
		3	M3_H8	5E-08	FF339601
		4	Contig68	1E-05	FF339598
		5	M14_C3	1E-03	FF339602
Fibrinogen	IPR002181	1	M4_G8	1E-14	EE743581
		2	M6_H11	4E-09	EE743583
		3	Contig144	1E-42	EE743580
		4	M11_F3	4E-14	EE743584
		5	M14_H4	3E-05	FF339607
		6	M2_E7	4E-52	EE743582
		7	M23_H4	4E-07	FF339610
von Willebrand factor, type A	IPR002035	1	M20_A3	6E-12	FF339611
		2	M23_F10	1E-03	FF339612
		3	M6_C2	3E-06	FF339613
von Willebrand factor, type D	IPR001846	1	M23_H6	5E-05	FF339614
Thrombospondin, type I	IPR000884	1	M18_H1	1E-03	FF339616
		2	M21_E6	1E-03	FF339617
		3	M7_F12	1E-05	FF339619
Protease inhibitor I4, serpin	IPR000215	1	M7_G3	2E-32	EE743622
		2	M7_A4	3E-19	EE743625
		3	M11_F4	2E-11	EE743621
Protease inhibitor I8	IPR002919	1	M20_E3	4E-08	EE743654
Peptidase S1 and S6, chymotrypsin/Hap	IPR001254	1	Contig121	8E-16	EE743596
or Peptidase, trypsin-like serine and cysteine	IPR009003	2	Contig177	3E-24	EE743606
		3	Contig179	4E-20	EE743603
		4	Contig182	7E-48	EE743601
		5	Contig22	1E-18	EE743607
		6	Contig85	8E-23	EE743598
		7	M1_A7	3E-10	FF339595
		8	M18_B11	2E-15	EE743605
		9	M12_D5	3E-18	EE743594
		10	M2_C9	5E-19	EE743608
		11	M20_F12	6E-21	EE743595
		12	M22_F8	2E-13	EE743599
		13	M22_G7	5E-18	FF339596
		14	M23_H5	4E-06	EE743597
		15	M3_B6	8E-22	EE743604
EGF-like	IPR006209	1	Contig143	1E-06	FF339637
or EGF calcium-binding	IPR013091	2	Contig15	3E-06	FF339638
		3	M13_A5	4E-07	FF339639
		4	M19_D1	2E-07	FF339640
		5	M7_B1	7E-05	FF339641
Proteinase inhibitor I2, Kunitz metazoan	IPR002223	1	M5_A7	1E-23	FF339642
Aspartic acid and asparagines hydroxylation site	IPR000152	1	M7_B1	8E-05	FF339641
Transglutaminase, C-terminal	IPR008958	1	M16_D7	1E-04	FF339643
MAM	IPR000998	1	M13_E4	5E-38	FF339644

**Figure 6:**
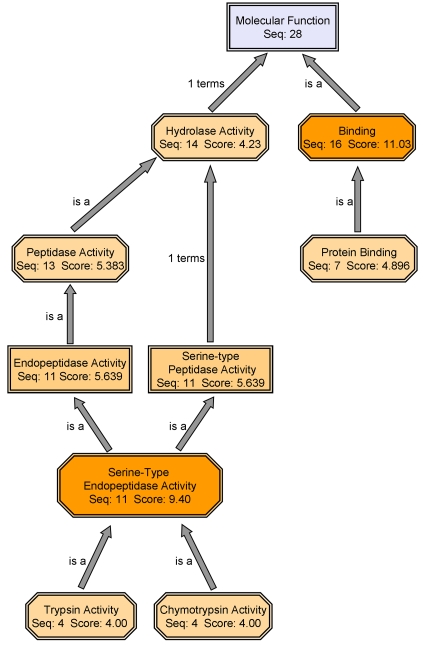
Gene ontology (GO) enrichment of coagulation-related transcripts during allogenic rejection. Twenty-eight significant “molecular function” sub ontology terms were identified by using the blast2go internet software. These GO terms were sorted into two major categories, the “serine-type endopeptidase activity” (belonging to the hydrolase activity group) and “binding” category. Different levels of orange hues indicate activity level according to GO term analysis.

All coagulation related sequences (including true orthologues and coagulation domains containing sequences) were subjected to mapping and annotation procedures using blast2go internet software (http://www.blast2go.de/). Gene ontology (GO) chart of twenty-eight significant “molecular function” sub ontology terms (Fig. 6) consist of two major categories: “serine-type endopeptidase activity” (belonging to the hydrolase activity group) and “binding”. The serine-type endopeptidase activity consists of mainly trypsin and chymotrypsin activities (eight terms in total).

**Figure 7:**
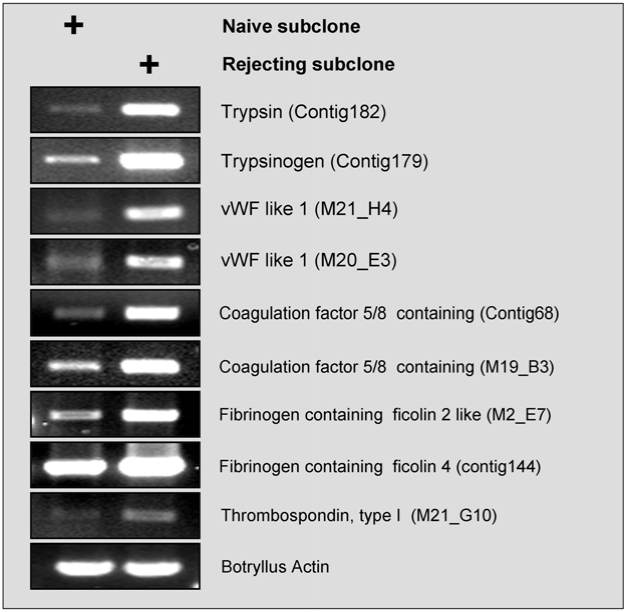
RT-PCR for selected gene transcripts found in the EST library (14). Assays were performed on cDNA prepared from mRNA collected from alloimmune-rejecting genotypes and their naïve counterparts.

In order to confirm the up-regulation of coagulation related genes during *Botryllus* rejection processes, we chose nine ESTs with high similarity to database coagulation proteins, preferably ESTs that exhibited similarity to both, coagulation proteins and coagulation domains (Table 4). RT-PCR reactions on independent RNA from immune unchallenged and rejecting *Botryllus* colonies, identified that all nine ESTs selected were differentially amplified (Fig. 7).

**Table 4:** Protein and motif matches of the sequences used in RT PCR.

EST ID	Protein match name	E-value	Motif match name	E-value
Contig182	Trypsin [*P. misakiensis*]	2E-73	Peptidase, trypsin-like serine and cysteine (IPR009003)	7E-48
Contig179	Tripsinogen [*B. schlosseri*]	3E-43	Peptidase, trypsin-like serine and cysteine (IPR009003)	4E-20
M21_H4M	von Willebrand Factor like 1 [*C. intestinalis*]	6E-40	—	—
20_E3	von Willebrand Factor like 1 [*C. intestinalis*]	1E-26	Protease inhibitor I8	4E-08
Contig68	milk fat globule-EGF factor 8 [*X. tropicalis*]	6E-10	Coagulation factor 5/8 (IPR000421)	1E-05
M19_B3	echinonectin [*S. purpuratus*]	8E-08	Coagulation factor 5/8 (IPR000421)	3E-12
M2_E7	ficolin 2 [*H. roretzi*]	4E-45	Fibrinogen (IPR002181)	4E-52
contig144	ficolin 4 [*H. roretzi*]	2E-42	Fibrinogen (IPR002181)	1E-42
M21_G10	Thrombospondin 2 [*H. sapiens*]	9E-07	Thrombospondin, type I (IPR000884)	2E-10

### Expression pattern of fibrinogen and vWF

In order to identify in *B. schlosseri* cellular and tissue sites for the expression of coagulation orthologues, we chose transcripts of two ESTs, a ficolin orthologue containing a fibrinogen domain, also identified as fibrinogen orthologue (BFF) and vWF orthologue (BvWF). cDNA fragments were amplified and used as probes templates. *In situ* hybridization, performed on histological sections of naïve and alloimmune rejecting colonies, elucidated enhanced expression patterns specific to histoincompatible reactions as well as common expressions not augmented by immune reactions.

**Figure 8:**
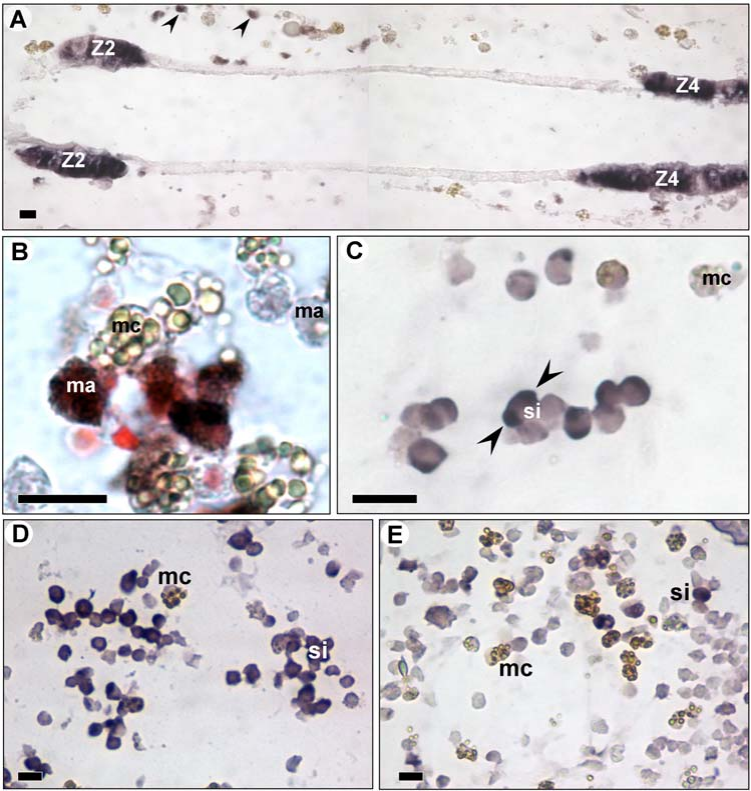
In-situ hybridization for BFF and BvWF. (A) Endostyle zones 2 (z2) and 4 (z4) stain positively with BvWF. Arrows indicate macrophage-like cells. (B) BvWF expressing macrophage-like cells in rejecting colonies (C) BFF staining in signet-ring blood cells. (D) Large aggregates of BFF- expressing signet-ring cells in interacting ampullae of rejecting colonies. (E) Few BFF- expressing signet-ring cells in ampullae of naive colonies. ma- macrophage-like cells, mc- morula cell, si- signet-ring cell, arrows head indicate intracellular staining. Scale bar: 10 µm.

BvWF *in situ* probe (320 bp sequence) was prepared based on the closest EST orthologue of the vertebrates vWF (EST id: M21_H4; Acc. No: EE743653). BvWF was found to be expressed in two major cell sites. One area of intense expression was restricted, in both, naïve and interacting colonies, to specific endostyle zones (Fig. 8, A), akin to the “endostyle zones” [22] numbers 2, 4 and mildly in zones 6 and 8 (not shown in the picture plane). This expression pattern coincides with that of *C. intestinalis* vWF orthologues [23]. The second specific BvWF expression was observed in a population of macrophage-like blood cells (Fig 8, A, B). This type of expression was exclusive to the interacting colonies and was rarely seen in naïve subclones. BvWF expressing macrophage-like cells were located mainly within-colonial vasculature and inner ampullae, indicating systemic colonial response to allogeneic challenges.

The second *in situ* probe (433 bp sequence) was prepared based on BFF cDNA (id: M2_E7; Acc. No: EE743582). BFF was confined to vacuoles within a specific subpopulation of circulating blood cells, signet ring cells in sizes of 5–9 µm (Fig. 8, C–E). BFF expressing cells were observed in the interacting ampullae of rejecting *Botryllus* colonies in significantly higher numbers compared to corresponding naïve sub-clones (Fig. 8D, E). Typically, most of these cells were attached to each other, forming small aggregates (Fig. 8D), a phenomenon which was not recorded in any of the naïve subclones (Fig. 8E).

### Immunohistochemistry for fibrinogen

**Figure 9:**
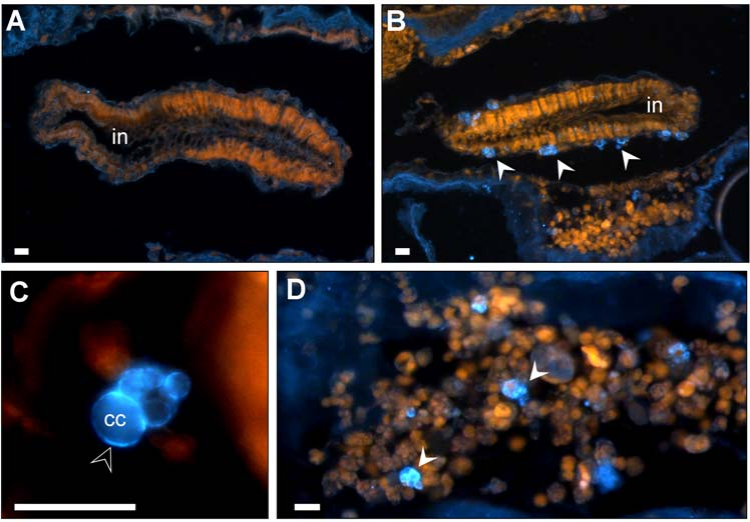
Immunostaining of fibrinogen. (A) Rabbit serum control. *Botryllus* section reacted with non-immune rabbit serum showing no specific staining. (B) *Botryllus* section reacted with a rabbit anti-human fibrinogen antiserum. Arrows indicate specifically stained fluorescent compartment cells around the intestine. (C) Enlargement of a compartment cell with membrane immunostaining. (D) Immunostained compartment cells in hemocoel (white arrows). cc- compartment cell, in- intestine, white arrow heads- compartment cell, black arrow head- membrane positive stain, scale bars: 10 µm.

Immunostaining for fibrinogen using anti human polyclonals, revealed, in naïve and immune challenged colonies alike, specific antibody affinity to a small population of *Botryllus* compartment cells, of 8–12 µm sizes. The stained cells were primarily located around the intestine (Fig. 9B) and scattered randomly in the animal's vasculature surrounding the hemocoel and the hemocoel itself (Fig. 9D). No specific localization of fibrinogen was assigned to the interacting ampullae and apparently not in the POR itself (we had difficulties in tracing specific coloring in the POR due to the unspecific coloring of the tunic surrounding it). Since the fluorescence dye illuminated primarily the cells' membranes (Fig. 9C), it is possible that the protein is membrane bound.

## Discussion

This study substantiates, molecularly, physiologically and morphologically, the existence and activation of vertebrates-like blood-based coagulation components in allorejections of the urochordate *Botryllus schlosseri*. Histoincompatible reactions in this species entail a wide genome expression, with a prominent representation of immune genes, as reflected by an EST library developed from rejecting *Botryllus* colonies [14]. Botryllid ascidians histoincompatibility leads to physical separation of the interacting partners through vasculature bleeding and the formation of necrotic clots (PORs; 24) that morphologically resemble vertebrates' clots (although the cells participating are different). These clots grow in the tunic matrix (in type-I rejection), within vasculature (type-II rejection) or along ruptured vasculature epithelium (Type-III rejection). Irrelevant to their size, all clot types involve the active destruction of blood cells, particularly of morula cells that are activated through the expression of the phenoloxidase system (PO; 12), not found in vertebrates. In addition to cell destruction, extensive deposition of fibrous ‘threads’ cross over the surrounding transparent tunic milieu, physically connecting newly formed PORs with tunic edges.

In arthropods and insects, the PO cascade participates in hemolymph coagulation, resulting from injury and bacterial elicitors, in addition to the functions of hemocytes in coagulation and melanization [9], [25]. Ascidians, while acquiring the PO system, are believed to induce only opsonization and cytotoxicity [12], but not coagulation [4], [13]. The absence of PO clotting properties in *Botryllus* is supported by the molecular data of *Botryllus* rejection library, consisting of no PO cascade elements, compared to the well-studied horseshoe crab system [8]. It is important, however, to note that key defense machineries (haemolymph coagulation, phenoloxidase-mediated melanization, lectin, complement, agglutinin response, reactive oxygen and nitrogen species production, phagocytosis, Toll- and Imd-linked AMP production) have remained conserved, opted for variable employments in all invertebrate phyla so far investigated [26]. Studies [11], [19], [24] revealed that clot formation in botryllid ascidians is not associated with vasculature injury or keeping bacteria away off the hemocoel. Nevertheless, the molecular analysis that we have conducted for the over expressed rejection genes has identified fundamental components associated with the activation of a vertebrates' coagulation system. This had been overlooked in former studies as orthologues genes play vital roles in other biological properties as well. Thrombospondin, first isolated from platelets that had been stimulated with thrombin [27], may serve as a representative example (Tables 2). While thrombospondins are known to interact with blood coagulation and anticoagulant factors, they participate actively in cell adhesion and proliferation, tissue repair, angiogenesis, as well as tumor metastasis [28]. Results [29] showed that CD36, a component of the thrombospondin receptor is highly expressed on *Botryllu*s phagocytes during the takeover phase of blastogenesis, revealing multifunctional properties of this molecule in basal chordates.

**Figure 10:**
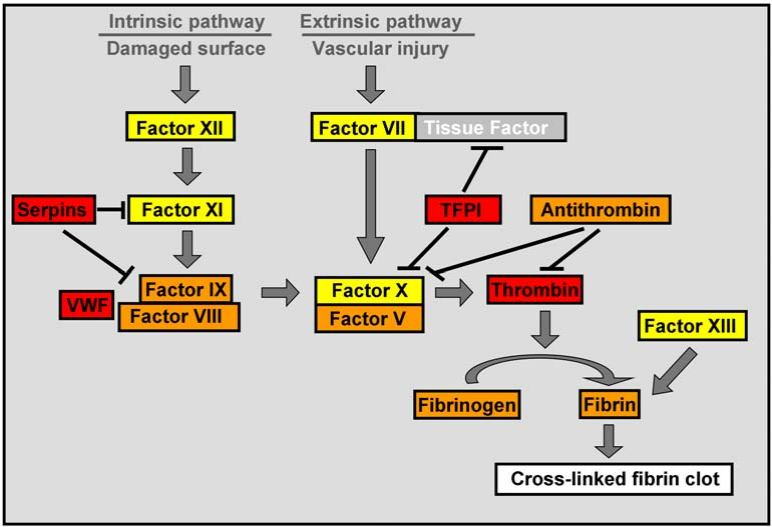
Relationship between *Botryllus* immune associated genes and human coagulation flow chart components. In red- true orthologues or molecules with proven activity. In orange- paralogues or domain containing components. In yellow- members of the same protein family (i.e. serine proteases/trypsin-like proteins). In gray- components that were not identified in *Botryllus* rejection library.

Following the 34 protein matches for cell adhesion and coagulation orthologues [14], the present study identified additional coagulation orthologues in the rejecting EST library and demonstrated the specific expression of nine coagulation orthologue transcripts in *Botryllus* rejection processes (including specific *in situ* hybridizations of two transcripts). Furthermore, 47 different coagulation-related domains were identified, altogether validating the wide expression of coagulation elements in botryllid ascidians immunity (Fig. 10). The human coagulation system consists of two major pathways. Both, contact activation pathway (intrinsic pathway) and tissue factor pathway (extrinsic pathway), consist of series of serine proteases reactions. Serine proteases, especially from the trypsin family, are highly represented among *Botryllus* rejection EST library ortholgues and its “molecular function” gene ontology analysis (Fig. 6). Trypsins, familiar participants in invertebrates and vertebrates digestive system enzymes, play a part in coagulation induction [30] and trypsin inhibitors inhibit coagulation [31]. In the ascidian *H. roretzi* trypsin induces blood cell aggregation [6]. Based on molecular data and the known effects of trypsin and trypsin inhibitors on coagulation, we suggest that members of the library trypsins are homologues to vertebrates proteolytic coagulation factors (equivalent to human factors VII and IX to XIII) and play a role in clot formation during botryllid ascidians' rejection processes.

A central serine protease proteolitic reaction in the coagulation cascade is the activation of coagulation factor X and the generation of thrombin (coagulation factor II) from a prothrombin precursor by factor Xa and its cofactor Va, which in turn catalyzes the conversion of the soluble plasma fibrinogen into insoluble fibrin. A true thrombin orthologue was identified in the *Botryllus* EST library along with two major thrombin inhibitors, antithrombin III (ATIII) and Tissue Factor Pathway Inhibitor (TFPI). The activity of ATIII is accelerated in the presence of optimum levels of heparin. Our results show that the typical clots of *Botryllus* were not developed in 25% of the heparin treated assays, although morula cells went through PO activation and melanization. This may be due to inhibition of the thrombin-based clotting activity. The partial impact of heparin treatment is probably associated with the type of rejection. The most common type-1 clots are formed in the tunic, with no damage to the blood vessel's walls, thus a blood born substance such as heparin may not be effective. Furthermore, results from the solitary ascidian *Styela plicata*, support potential roles of ascidian-borne heparin in coagulation. A heparin-like substance that was isolated from this species [32] showed 10% anticoagulant potency compared to mammalian counterpart and 5% potency in thrombin inhibition activity [33]. Similarly, dermatan sulfates with anticoagulant activity were described in the ascidians *S. plicata* and *H. pyriformis* [34].

Fibrinogen, a precursor of fibrin and the clot-forming molecule, was absent in the first blast matches list of *Botryllus* EST library, as in other invertebrates genomes [4], [5]. Nevertheless, secondary blast matches revealed fibrinogen and several fibrinogen motifs in previously designated ficolin orthologues. Ficolins are familiar pattern recognition receptors (PRRs) and initiators of the lectin complement pathway, and because of their similarity to fibrinogen it was suggested that both genes had derived from a common ancestor [35]. *In-situ* hybridization for BFF showed amplified expressions within signet-ring cells, aggregated inside interacting ampullae, supporting its relevancy to *Botryllus* rejection process. Interestingly, immunohistochemistry for human fibrinogen indicated the ubiquitous existence of a protein with high affinity to human fibrinogen antibody in *Botryllus* compartment cells, the derivatives of the signet-ring cell lineage [36], [37]. This corresponds with onset of protein production. First, transcription in precursor blood cells (signet-ring), located in the interacting blood vessels, then translation in mature blood cells (compartment cells) located around the intestine and inner organs. Signet-ring cells and compartment cells are also known to be associated with the formation of the rejection clot (POR; 11)

In conclusion, this study demonstrates the existence and specific expression of orthologue components of the mammalian coagulation network (summarized in Fig. 10) in an invertebrate urochordate that lacks an ‘injury triggered’ clotting system and suggests their involvement in innate immune allorejection responses. The effector arm of allorecognition in this species, therefore, reveals the efficient use of existing vertebrates clotting architectures in a different biological feature. Further studies are needed to elucidate biological properties of coagulation related genes in urochordate allorecognition. However, the notion that the complex gene organizations and protein structures of the mammalian coagulation system have evolved from the reduplication and functional diversification of few ancestral genes [2], [4], [38], provides an evolutionary importance to the roles of these genes in the biological properties of basal chordates. In addition, such a study may contribute to better understanding of the evolution of the coagulation system and to insights into mammalians' genomes evolution. This will enable molecular dissection of the pathways involved in the urochordate innate immunity. Coagulation and innate immunity, not only are highly integrated [1], [39], exhibiting crosstalk features (i.e., complement and coagulation associations; 40) but may share an ancestral molecular stem early in eukaryotic evolution.

## Supporting Information

Video S1 Untreated Botryllus colony showing normal heart beats. Fifteen heart beats were recorded during the time length of 10 seconds as captured in the clip.
(1.68 MB MPG)

Video S2 Treated Botryllus colony, five minutes after administration of 40,000 units/L Heparin in sea water. Only eight heart beats were recorded for the same heart (in the center) during the same time length as in Video S1.
(1.67 MB MOV)
